# Study design and data analysis considerations for the discovery of prognostic molecular biomarkers: a case study of progression free survival in advanced serous ovarian cancer

**DOI:** 10.1186/s12920-016-0187-4

**Published:** 2016-06-10

**Authors:** Li-Xuan Qin, Douglas A. Levine

**Affiliations:** Department of Epidemiology and Biostatistics, Memorial Sloan Kettering Cancer Center, New York, NY 10065 USA; Department of Surgery, Memorial Sloan Kettering Cancer Center, New York, NY 10065 USA

## Abstract

**Background:**

Accurate discovery of molecular biomarkers that are prognostic of a clinical outcome is an important yet challenging task, partly due to the combination of the typically weak genomic signal for a clinical outcome and the frequently strong noise due to microarray handling effects. Effective strategies to resolve this challenge are in dire need.

**Methods:**

We set out to assess the use of careful study design and data normalization for the discovery of prognostic molecular biomarkers. Taking progression free survival in advanced serous ovarian cancer as an example, we conducted empirical analysis on two sets of microRNA arrays for the same set of tumor samples: arrays in one set were collected using careful study design (that is, uniform handling and randomized array-to-sample assignment) and arrays in the other set were not.

**Results:**

We found that (1) handling effects can confound the clinical outcome under study as a result of chance even with randomization, (2) the level of confounding handling effects can be reduced by data normalization, and (3) good study design cannot be replaced by post-hoc normalization. In addition, we provided a practical approach to define positive and negative control markers for detecting handling effects and assessing the performance of a normalization method.

**Conclusions:**

Our work showcased the difficulty of finding prognostic biomarkers for a clinical outcome of weak genomic signals, illustrated the benefits of careful study design and data normalization, and provided a practical approach to identify handling effects and select a beneficial normalization method. Our work calls for careful study design and data analysis for the discovery of robust and translatable molecular biomarkers.

**Electronic supplementary material:**

The online version of this article (doi:10.1186/s12920-016-0187-4) contains supplementary material, which is available to authorized users.

## Background

Accurate discovery of molecular biomarkers that are prognostic of a clinical outcome is an important yet challenging task [1]. A main reason for the difficulty is the combination of the typically weak signal for a clinical outcome and the frequently strong noise due to microarray handling effects [2]. In particular, array handling effects can increase data variability and often confound with the outcome of interest, which have been reported profoundly in high-throughput genomic studies as a reason for dubious or even erroneous findings [3].

To account for handling effects in microarray studies, careful study design has been advocated and data normalization has been routinely used for discovering molecular markers that can distinguish two or more sample groups [4–7]. We recently conducted a proof-of-principle study on the feasibility and benefits of careful study design (that is, uniform experimental handling and balanced array-to-sample-group assignment via the use of blocking and randomization) for biomarker discovery in clinical microarray studies [8–10]. We generated two microRNA (miRNA) array datasets for the same set of tumor samples (96 advanced serous ovarian cancer and 96 endometrioid endometrial cancer tumors): arrays in one dataset were collected with careful study design, while arrays in the other dataset were not [11, 12]. As a proof of concept, we assessed the benefits of study design, in comparison with post-hoc data normalization, when the outcome is tumor type, whose level of signal is relatively strong. Through both empirical analysis and re-sampling based simulations, we showed that careful study design can more effectively improve the accuracy of biomarker discovery than data normalization. It remains to be elucidated what roles study design and data normalization can play for the discovery of prognostic biomarkers for a survival outcome especially when its level of signal is weak.

In this paper, we took progression free survival (PFS) in advanced serous ovarian cancer as an example and assessed the role of study design and data normalization on prognostic biomarker discovery, using the ovarian cancer data from the pair of array datasets that we have previously collected. We found that (1) handling effects can confound the outcome of interest as a result of chance even when randomization was used for array assignment, (2) the level of handling effects can be partially reduced by post-hoc data normalization, and (3) while useful to certain extent data normalization cannot replace the use of good study design for data collection. These findings showcased the difficulty of finding prognostic biomarkers for a clinical outcome of weak signal, illustrated the benefits of careful study design and data normalization for accurate discovery of prognostic biomarkers, and underscored the importance of checking for evidence of confounding handling effects even in the presence of randomization. Comparing with our previous works on the paired datasets, the novel contributions of this paper are (1) the examination of a weak yet clinically meaningful survival endpoint, (2) the study of using only randomization and no blocking for data collection, and (3) the development of a new and practical approach for detecting handling effects and assessing a normalization method.

## Methods

Human tumor tissues used in this study were obtained from participants who provided informed consent and their use in our study was approved by the Memorial Sloan Kettering Cancer Center Institutional Review Board.

### Data collection

A set of 192 untreated primary gynecologic tumor samples (96 endometrioid endometrial tumors and 96 serous ovarian tumors) were collected at Memorial Sloan Kettering Cancer Center during the period of 2000–2012. The samples were profiled using the Agilent Human miRNA Microarray (Release 16.0), following the manufacturer’s protocol. This array platform contains 3,523 markers (representing 1,205 human and 142 human viral miRNAs) and for each marker multiple replicates (ranging from 10 to 40). In addition, it has eight arrays on each glass slide (that is, the experimental ‘block’) arranged as two rows and four columns. Two datasets were originated from the same set of samples using different processes of array-to-sample assignments and experimental handling. The first dataset was created using randomization and blocking in the array-to-sample assignment and was handled by one experienced technician in one experimental run. Here, blocking means that arrays on each block are assigned proportionally to each tumor group, and randomization means that within each tumor group arrays are randomly paired with samples. The second dataset used an array assignment in the order of tumor sample collection and was handled by two technicians in multiple runs. In this study, we used the portion of the data for the 96 serous ovarian tumor samples, for which only randomization and no blocking were used for array assignment. More details on data collection can be found in Qin et al. [11].

### Array data preprocessing

We preprocessed the array data using log2 transformation and median summarization for replicates of the same marker on the array. The randomized array dataset was analyzed both with and without quantile normalization; the un-randomized array dataset was analyzed with quantile normalization [12]. When quantile normalization was used, it was applied after log2 transformation and before median summarization [13].

### Survival analysis

Progression free survival was calculated as the time interval from primary tumor resection to progression, death, or loss of follow up, whichever occurs first. Association between clinical and molecular covariates with PFS was assessed with the Cox regression model and the score test [14]. Alternatively, PFS was also dichotomized at its median (18 months) and association between molecular covariates and PFS at 18 months was assessed using the *t*-test statistic comparing the two PFS groups. The two-sided *p*-value was calculated. A p-value cutoff of 0.05 was used as the significance cutoff for clinical variables and 0.01 for molecular markers.

### Definition of negative and positive control markers for detecting handling effects

For the Agilent miRNA array, we defined poorly-expressed markers as those with mean expression below a small cutoff (preprocessed data <6) reflecting little biological effects and mainly handling effects, and well-expressed markers as those with mean expression above a cutoff (preprocessed data >8) reflecting mainly biological effects. We used the cutoff of mean expression 6 to select poorly-expressed markers because it was close to the low end of the dynamic range of Agilent arrays and the selected markers also had a very narrow range of expression level with the standard deviation ranging roughly from 0.1 to 0.5. The randomized dataset had 217 well-expressed markers belonging to 133 miRNAs, among which 84 were represented by two well-expressed markers and 49 by one. Pearson correlation coefficients were calculated between replicate markers for each of the 84 miRNAs. The randomized dataset had 2805 poorly-expressed markers representing 1070 miRNAs, among which 331 were represented by four poorly-expressed markers, 89 by three, 564 by two, and 86 genes by one. One single poorly-expressed marker was randomly selected for each miRNA represented by multiple poorly-expressed markers. Pairwise Pearson correlation coefficients were calculated among the 1070 poorly-expressed markers representing 1070 unique miRNAs.

## Results

### Analysis of clinical characteristics

**Fig. 1:**
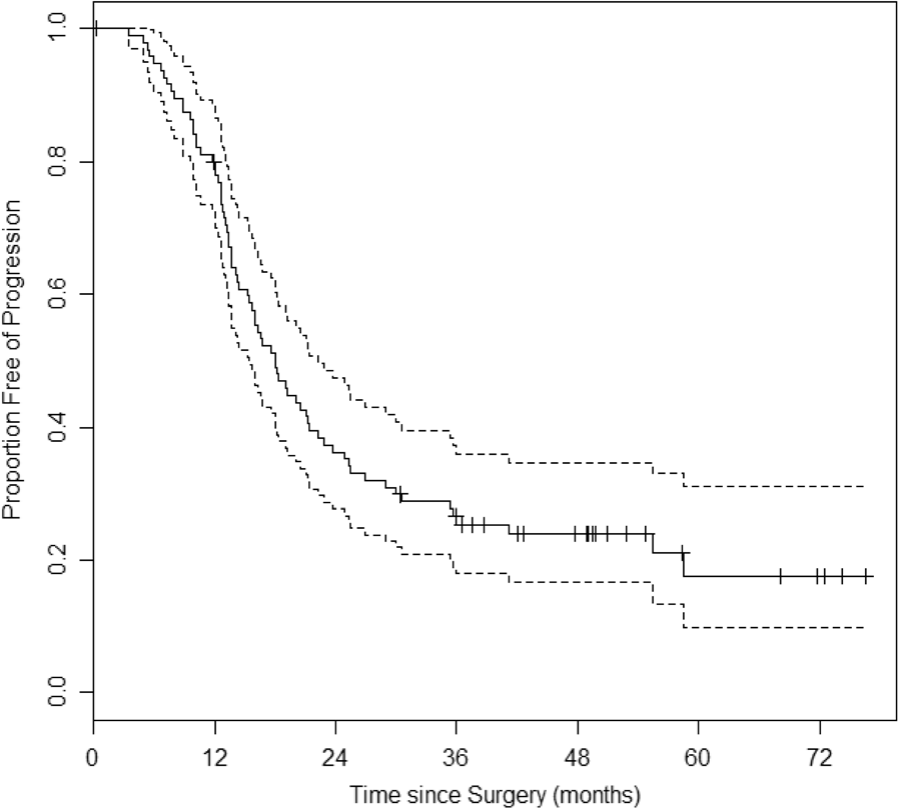
KM curve for PFS among the 96 ovarian cancer patients

Table 1 lists the clinical characteristics of the 96 primary high-grade ovarian cancer patients in our study. Among these patients, 67 (70 %) were stage III, 29 (30 %) stage IV, and 43 (45 %) had no residual disease; the median PFS was 18 months (95 % CI: 15 ~ 21 months) and the median follow up among progression free survivors was 49 months (Fig. 1). In agreement with the literature, tumor stage (*p =* 0.03) and residual disease (*p <* 0.01) were both significant prognostic variables for PFS.

**Table 1:** Patient characteristics among the 96 ovarian cancer samples

	*N* (%)	Median PFS in months (95 % CI)	*P*-value
All	96	18.0 (15.3–21.4)	
Age <60	41 (43 %)	22.2 (13.7–35.4)	0.25
> =60	55 (57 %)	17.1 (13.6–20.5)	
Stage III	67 (70 %)	20.0 (16.3–30.0)	0.03
IV	29 (30 %)	14.4 (12.7–19.2)	
Residual disease 0 cm	43 (45 %)	28.9 (21.1–55.5)	<0.01
> 0 cm	53 (55 %)	14.1 (12.1–16.5)

### PFS analysis of the randomized array data

**Fig. 2:**
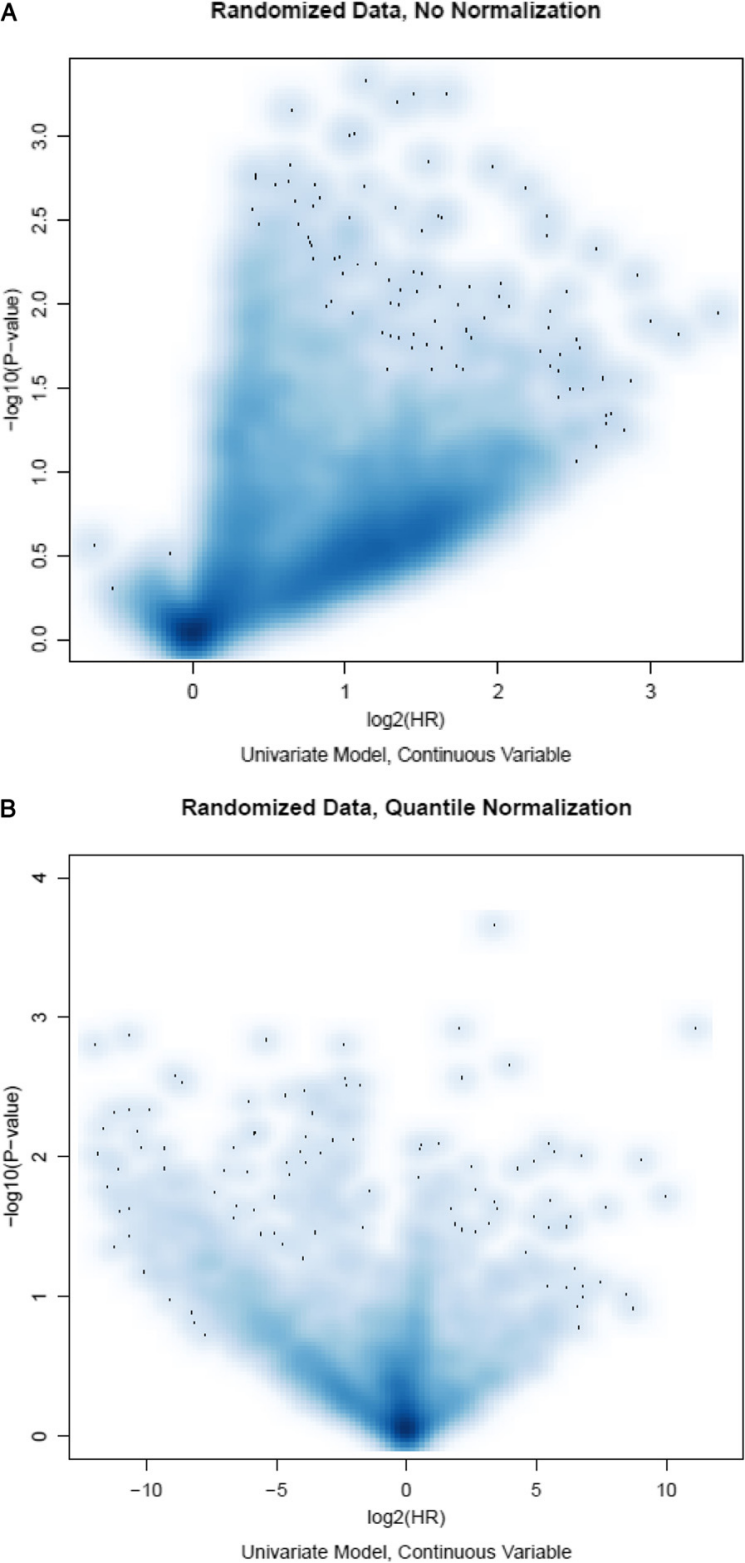
Volcano plot for the PFS analysis in the randomized data before (**a**) and after (**b**) quantile normalization

We assessed the association of miRNAs with PFS in ovarian cancer using the randomized dataset, which was collected with uniform handling and randomized assignment of arrays to samples (Additional file 1: Figure S1). Surprisingly, the vast majority of miRNAs had a hazard ratio greater than one, indicating positive associations with risk to progression (Fig. 2a). This observation is independent of the analysis method used, as the same pattern persists when the analysis is done by dichotomizing PFS at its median 18 months and comparing those who progressed at 18-month and those who did not using a two-sample *t*-test (Additional file 1: Figure S2); it is also independent of the scale of the array data such as dichotomization (Additional file 1: Figure S3) or the adjustment for tumor stage and residual disease (Additional file 1: Figure S4).

**Fig. 3:**
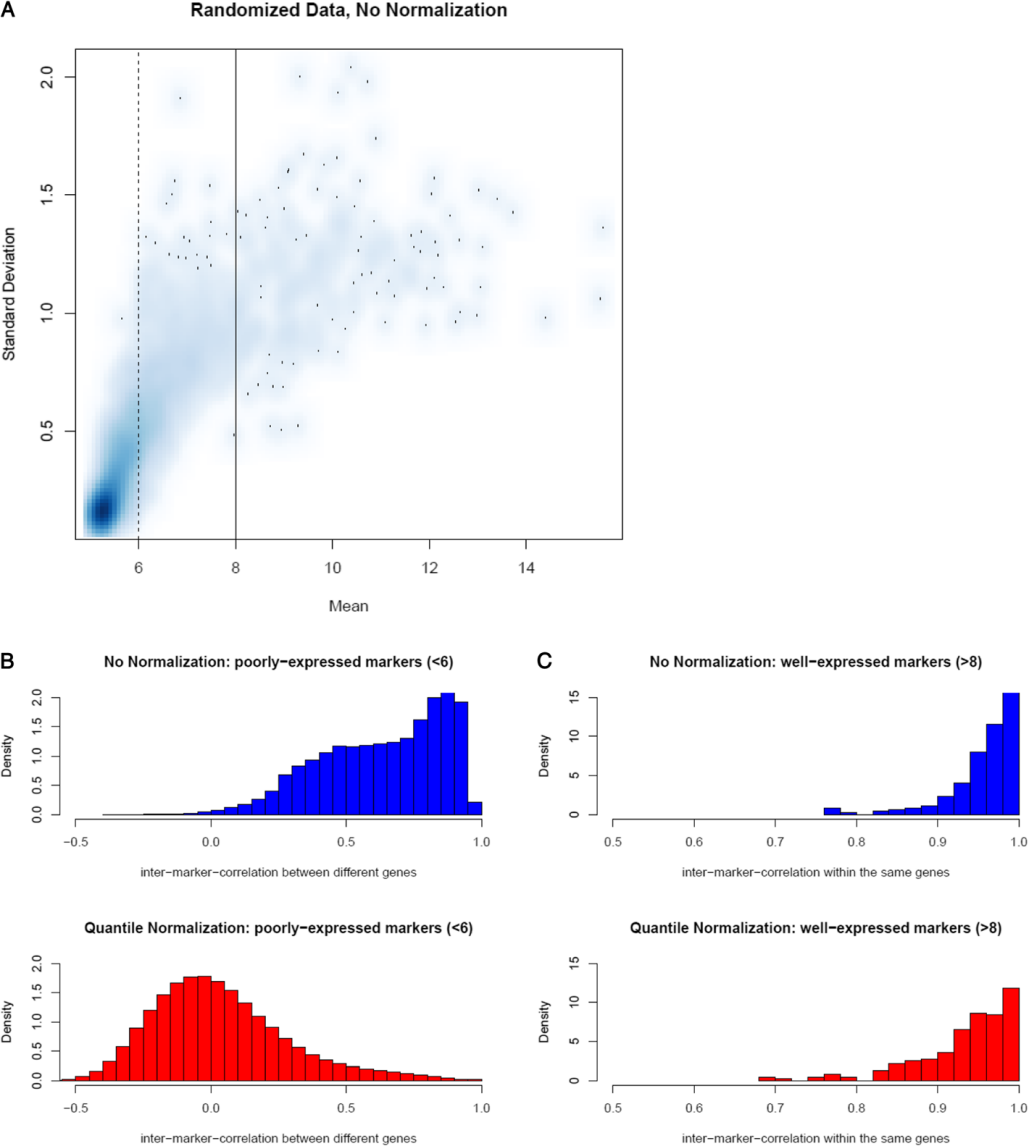
**a** Scatter plot of marker-specific standard deviations versus marker-specific means for the selection of well-expressed and poorly-expressed miRNAs. **b** Histogram of the Pearson correlation coefficients among the collection of unique markers for each poorly-expressed miRNA, before (top panel) and after (bottom panel) quantile normalization. **c** Histogram of the Pearson correlation coefficients calculated among replicate markers for each well-expressed miRNA, before (top panel) and after (bottom panel) quantile normalization

We seek to understand whether the predominance of positive risk association is due to true biological signals or confounding handling effects (despite randomization and uniform handling). Towards this end, we defined *poorly-expressed* markers as those with mean expression below a small cutoff reflecting few biological effects and primarily handling effects, and *well-expressed* markers as those with mean expression above a cutoff reflecting primarily biological effects and some handling effects (Fig. 3a). Our reasoning for diagnosing the existence of handling effects using poorly-expressed and well-expressed markers is as follows. If handling effects exist in the data, they manifest high positive correlations among the collection of poorly-expressed markers (one single marker kept for each represented miRNA) simply due to the similar handling effects shared among markers; however, these positive correlations should dissipate after the data are normalized as an effort to remove handling effects. In contrast, high positive correlation should exist between replicate markers for each well-expressed miRNA (that is represented by more than one marker) due to both shared biological signals and shared handling effects and would persist even after normalization. Figure 3b and c show the correlation coefficients among the poorly-expressed markers (more specifically, the collection of one single poorly-expressed marker for each represented miRNA) and those between replicate well-expressed markers for each represented miRNA. The former peaked towards one before normalization and centered around zero after normalization, while the latter was nearly one both before and after normalization. As suggested by a reviewer, we also examined the *p*-value distribution among poorly-expressed markers and observed a shift towards the uniform distribution after normalization (Additional file 1: Figure S5).

The aforementioned findings collectively suggest that, (1) even with uniform handling, the randomized dataset was not completely free of handling effects, which may reflect an inherent and unavoidable nature of high-throughput data, and (2) despite randomized array-to-sample assignment, handling effects can still confound with the outcome as a result of chance. Therefore the predominantly positive risk association among the miRNAs was likely due to handling effects rather than biological signals.

### Normalization to adjust for confounding handling effects

When evidence of confounding handling effects is observed, one should consider the use of data normalization before any further analysis. A beneficial normalization should maximally remove handling effects while minimally impact the biological effects [15]. This can translate to reducing the high correlation among unique poorly-expressed markers to around zero and at the same time keeping the high correlation among replicate probes for each well-expressed markers intact, which was what we have observed for quantile normalization, a most commonly used method for microarray data normalization (Fig. 3b and c). In contrast, median normalization was less effective in removing the correlation between poorly-expressed markers (Additional file 1: Figure S6).

We re-analyzed the randomized data for PFS association after quantile normalization. As a result of normalization, the numbers of positive and negative risk-associated markers were more evenly distributed (Fig. 2b). Two highly-expressed markers, both representing miR-23a, were significantly associated with PFS (*p =* 0.006 and HR = 1.5 for one marker; *p =* 0.007 and HR = 1.4 for another) (Additional file 1: Figure S7), consistent with recent reports showing that miR-23a promoted tumor progression in multiple cancer types [16–18].

We note that, although useful to some extent, normalization cannot replace good study design. In our study, good study design refers to uniform handling and random array-to-sample assignment. In a second array dataset on the same 96 ovarian cancer samples where no careful study design was exercised, no well-expressed markers were identified to be significantly associated with PFS even with quantile normalization (Additional file 1: Figure S8). In particular, the two markers of miR-23a were no longer significant (*p =* 0.19 and *p =* 0.23).

## Discussion

Our data have demonstrated that, despite uniform handling and randomization, there can still be confounding handling effects in the data, which could be detrimental to the biomarker discovery for weak clinical outcomes. Our work strongly supports the practice that (1) when the outcome of interest is known at the time of array generation, one should use blocking or stratification to further balance handling effects and hence avoid their confounding effects (In fact, many array platforms come as natural ‘blocks’: for example, the Illumina mRNA array platforms have six, eight, or twelve arrays on each glass slide (the block), and the Agilent miRNA array platform has eight arrays on each glass slide arranged as two rows and four columns.); (2) when blocking is not possible (for example, when the outcome of interest is unknown or when the outcome of interest is a secondary phenotype), one could use randomization in array assignment to reduce the chance of confounding handling effects; (3) even in the presence of randomization, one should still assess for evidence of confounding handling effects and if positive use data normalization before making any biological inference from the data.

We have presented a simple yet useful method for assessing the presence of handling effects. Our method is based on the selection of negative control markers that are expected to have no biological activities and positive control markers that share similar biological activities, and the assessment of the correlation structure among each set of control markers before versus after data normalization. For Agilent miRNA arrays, we have demonstrated the use of poorly-expressed markers as negative controls and well-expressed markers as positive controls. Although the results in this paper were based on the positive and negative control markers defined on the randomized data for the proof of concept, we have found that similar markers were selected based on the un-randomized data (Additional file 1: Table S9). Therefore, when only the un-randomized data is available in a study, one can still select the positive and negative markers using the un-randomized data. We further assessed the use of poorly-expressed markers and well-expressed markers in the miRNA array data from the Cancer Genome Atlas ovarian cancer study and observed similar change of correlation structure before versus after normalization, supporting the generalizability of our choice of the negative and positive control markers for the purpose of assessing the presence of handling effects (Additional file 1: Figure S10) [19].

## Conclusions

Our work in this paper is consistent with our previous study on the benefits of careful study design and data normalization, and it provides meaningful new information on the possibility of confounding handling effects even in the presence of randomization and a practical approach to check for such confounding handling effects. This work continues our advocacy of careful study design and data analysis in order to accurately discover robust and translatable biomarkers for clinical applications.

## Abbreviations

miRNA, microRNA; PFS, Progression Free Survival

## Additional file

Additional file 1: Figure S1 is a figure showing the boxplots for the 96 ovarian arrays in the randomized data. Additional file 1: **Figure S2** is a figure showing the volcano plot for PFS analysis in the ovarian randomized data when the outcome is PFS at 18-month. Additional file 1: **Figure S3** is a figure showing the volcano plot for PFS analysis in the ovarian randomized data when the array data is dichotomized. Additional file 1: **Figure S4** is a figure showing the volcano plot for PFS analysis in the ovarian randomized data when adjusting for stage and residual disease. Additional file 1: **Figure S5** has two figures showing the distribution of the p-values from PFS analysis among poorly-expressed markers and well-expressed markers. Additional file 1: **Figure S6** has histograms of the Pearson correlation coefficients and of the PFS p-values among poorly-expressed markers and among replicate markers for each well-expressed miRNA after median normalization for the randomized data. Additional file 1: **Figure S7** is a figure showing the Kaplan-Meier curve for miR-23a when its expression data were quantile normalized and dichotomized at the median. Additional file 1: **Figure S8** is a figure showing the volcano plot for PFS analysis using the ovarian un-randomized data with quantile normalization. Additional file 1: **Table S9** is a table comparing the well- and poorly-expressed markers selected by the randomized data versus those selected by the un-randomized data. Additional file 1: **Figure S10** has figures showing the selection of poorly-expressed and well-expressed markers and their correlation distribution before and after quantile normalization, using the miRNA array data from the Cancer Genome Atlas ovarian cancer study (*n =* 462).
