# Supplementary material for: Study design and data analysis considerations for the discovery of prognostic molecular biomarkers: a case study of progression free survival in advanced serous ovarian cancer
Source: BMC Med Genomics. 2016 Jun 10;9:27. doi: 10.1186/s12920-016-0187-4 (PMC4901402; doi:10.1186/s12920-016-0187-4)
Supplement: Additional file 1: Figure S1 — is a figure showing the boxplots for the 96 ovarian arrays in the randomized data. Additional file 1: Figure S2 is a figure showing the volcano plot for PFS analysis in the ovarian randomized data when the outcome is PFS at 18-month. Additional file 1: Figure S3 is a figure showing the volcano plot for PFS analysis in the ovarian randomized data when the array data is dichotomized. Additional file 1: Figure S4 is a figure showing the volcano plot for PFS analysis in the ovarian randomized data when adjusting for stage and residual disease. Additional file 1: Figure S5 has two figures showing the distribution of the p-values from PFS analysis among poorly-expressed markers and well-expressed markers. Additional file 1: Figure S6 has histograms of the Pearson correlation coefficients and of the PFS p-values among poorly-expressed markers and among replicate markers for each well-expressed miRNA after median normalization for the randomized data. Additional file 1: Figure S7 is a figure showing the Kaplan-Meier curve for miR-23a when its expression data were quantile normalized and dichotomized at the median. Additional file 1: Figure S8 is a figure showing the volcano plot for PFS analysis using the ovarian un-randomized data with quantile normalization. Additional file 1: Table S9 is a table comparing the well- and poorly-expressed markers selected by the randomized data versus those selected by the un-randomized data. Additional file 1: Figure S10 has figures showing the selection of poorly-expressed and well-expressed markers and their correlation distribution before and after quantile normalization, using the miRNA array data from the Cancer Genome Atlas ovarian cancer study (n = 462). [file 12920_2016_187_MOESM1_ESM.docx]

**Additional file 1**

**Figure S1.** The boxplots for the 96 ovarian arrays in the randomized data.

**
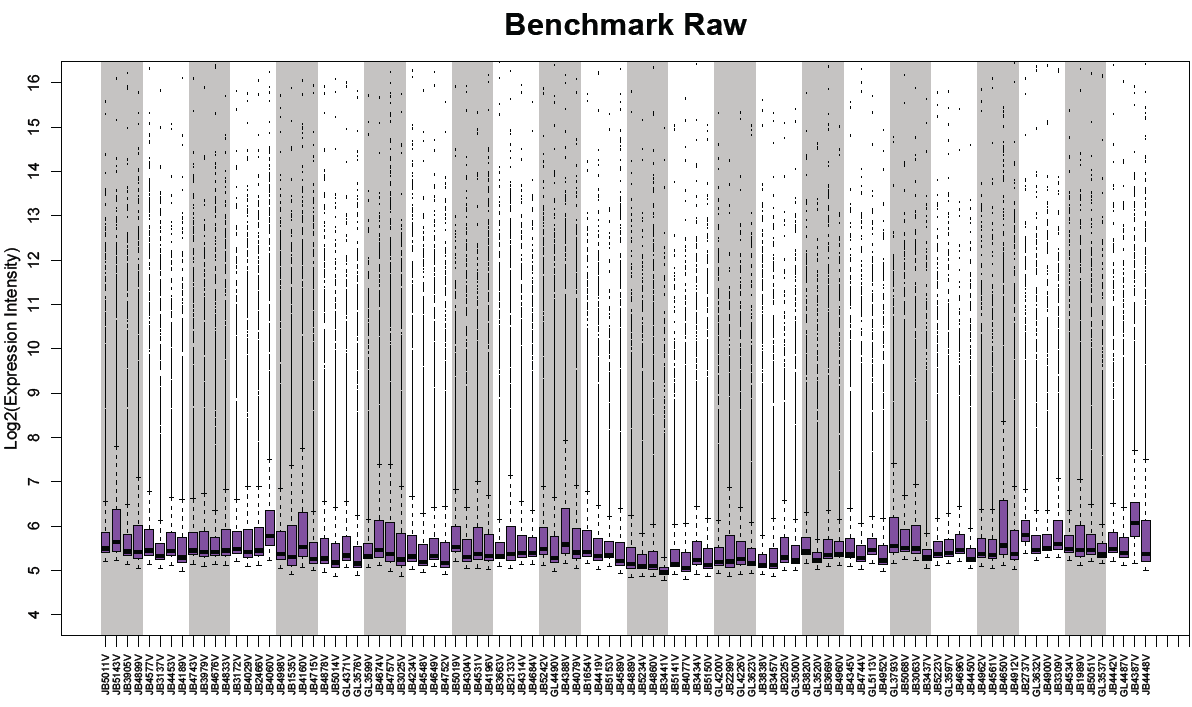
**

**Figure S2**. Volcano plot for PFS analysis in the ovarian randomized data when the outcome is PFS at 18-month.

**Figure S3**. Volcano plot for PFS analysis in the ovarian randomized data when the array data is dichotomized.

**Figure S4**. Volcano plot for PFS analysis in the ovarian randomized data when adjusting for stage and residual disease.

**Figure S5A**. Histogram of p-values for PFS analysis among poorly-expressed markers.

**Figure S5B**. Histogram of p-values for PFS analysis among well-expressed markers.

**Figure S6**. Left column: Histogram of the Pearson correlation coefficients among poorly-expressed markers (top left panel) and among replicate markers for each well-expressed miRNA (bottom left panel) after median normalization for the randomized data. Right column: Histogram of PFS p-values among poorly-expressed markers (top left panel) and among replicate markers for each well-expressed miRNA (bottom left panel) after median normalization for the randomized data.

**Figure S7**. Kaplan-Meier curve for miR-23a. Expression data were quantile normalized and dichotomized at the median. P-value=0.006 and HR=1.5.

**Figure S8**. Volcano plot for PFS analysis using the ovarian un-randomized data with quantile normalization.

**Table S9**. Comparison of well- and poorly-expressed markers selected by the randomized data versus those selected by the un-randomized data.

|  |  | **Un-randomized Data** | |
| --- | --- | --- | --- |
|  |  | **Well-expressed** | **Otherwise** |
| **Randomized Data** | **Well-expressed** | 207 | 10 |
|  | **Otherwise** | 3 | 3303 |

|  |  | **Un-randomized Data** | |
| --- | --- | --- | --- |
|  |  | **Poorly-expressed** | **Otherwise** |
| **Randomized Data** | **Poorly-expressed** | 2805 | 0 |
|  | **Otherwise** | 101 | 617 |

**Figure S10A.** Scatter-plot of marker-specific mean versus standard deviation for the TCGA ovarian miRNA array data (n=462).

**Figure S10B.** Histogram of inter-marker correlation coefficients among poorly-expressed miRNAs (with one unique marker per miRNA) for the TCGA ovarian miRNA array data (n=462).

**Figure S10C.** Histogram of inter-marker correlation coefficients between replicate markers for each well-expressed miRNA for the TCGA ovarian miRNA array data (n=462).
